# Automated Smart Home Assessment to Support Pain Management: Multiple Methods Analysis

**DOI:** 10.2196/23943

**Published:** 2020-11-06

**Authors:** Roschelle L Fritz, Marian Wilson, Gordana Dermody, Maureen Schmitter-Edgecombe, Diane J Cook

**Affiliations:** 1 College of Nursing Washington State University Vancouver, WA United States; 2 School of Nursing and Midwifery Edith Cowan University Joondalup Australia; 3 School of Electrical Engineering and Computer Science Washington State University Pullman, WA United States

**Keywords:** pain, remote monitoring, sensors, smart homes, multiple methods

## Abstract

**Background:**

Poorly managed pain can lead to substance use disorders, depression, suicide, worsening health, and increased use of health services. Most pain assessments occur in clinical settings away from patients’ natural environments. Advances in smart home technology may allow observation of pain in the home setting. Smart homes recognizing human behaviors may be useful for quantifying functional pain interference, thereby creating new ways of assessing pain and supporting people living with pain.

**Objective:**

This study aimed to determine if a smart home can detect pain-related behaviors to perform automated assessment and support intervention for persons with chronic pain.

**Methods:**

A multiple methods, secondary data analysis was conducted using historic ambient sensor data and weekly nursing assessment data from 11 independent older adults reporting pain across 1-2 years of smart home monitoring. A qualitative approach was used to interpret sensor-based data of 27 unique pain events to support clinician-guided training of a machine learning model. A periodogram was used to calculate circadian rhythm strength, and a random forest containing 100 trees was employed to train a machine learning model to recognize pain-related behaviors. The model extracted 550 behavioral markers for each sensor-based data segment. These were treated as both a binary classification problem (event, control) and a regression problem.

**Results:**

We found 13 clinically relevant behaviors, revealing 6 pain-related behavioral qualitative themes. Quantitative results were classified using a clinician-guided random forest technique that yielded a classification accuracy of 0.70, sensitivity of 0.72, specificity of 0.69, area under the receiver operating characteristic curve of 0.756, and area under the precision-recall curve of 0.777 in comparison to using standard anomaly detection techniques without clinician guidance (0.16 accuracy achieved; *P*<.001). The regression formulation achieved moderate correlation, with *r*=0.42.

**Conclusions:**

Findings of this secondary data analysis reveal that a pain-assessing smart home may recognize pain-related behaviors. Utilizing clinicians’ real-world knowledge when developing pain-assessing machine learning models improves the model’s performance. A larger study focusing on pain-related behaviors is warranted to improve and test model performance.

## Introduction

More than 50 million US adults suffer from chronic pain, and 19.6 million experience high-impact chronic pain severe enough to interfere with daily living or work activities [1]. Guidelines from the Centers for Disease Control and Prevention favor nonopioid strategies to manage pain [2]; however, professionals treating pain have raised concerns that reducing access to opioids for the 18 million Americans using them for chronic pain will cause needless suffering [3]. Thus, there is a critical need to better understand how adults experiencing pain manage their symptoms at home. Artificial intelligence (AI) may afford the opportunity for observations leading to new understandings and improved home-based pain management. AI for health care has already afforded new perspectives [4] on automated assessments leading to novel and timely interventions [5]. Machine learning (ML) models are used in medical imaging [6,7], neurology [8], cardiology [9,10], pulmonology [11], nephrology [12,13], gastroenterology [14], pathology [15,16], health care informatics [17,18], and clinical decision support [5,19]. ML models capable of automated in-home assessments and alerts are also in the early stages of supporting individualized home-based interventions [20-24].

Social cognitive theory supports that mastering daily tasks is key to living with chronic pain [25]. Pain interference is best captured through observing physical and social changes in daily activities [26]. However, most pain assessments occur in clinical settings. Advances in smart home technology provide the opportunity for unobtrusive and continuous monitoring of daily activities [27]. Such monitoring offers sensor-based observation of activities, routines, and behaviors and could provide direct evidence of clinically relevant changes in daily routines [21,22], sleep [28], and socialization [22]. To date, smart home ML models have proven capable of differentiating behavior markers between groups [29,30], modeling characteristics of older adults’ daily activities [20], recognizing dementia-related behaviors [31], predicting cognitive and mobility scores [20,32], forecasting behavior-based sleep and wake patterns [28], and recognizing health events such as falls [22], pneumonia [33], and depression [34].

A common limitation to developing ML health-behavior models is the time needed to provide real-world context (ground truth) for datasets. However, evidence exists that techniques accounting for small samples and low levels of consistent reporting can produce robust models [35,36]. While it may be difficult to predict pain experience trajectories, well-validated risk prediction models have identified individuals at risk for long-term pain [37]. Predictive models have been tested for low back pain [37], post-surgical cancer pain [38], and pain with dementia [39]. Statistical modeling has also been used to predict physical and psychological factors for long-term pain [40]; however, models have not yet been developed to identify pain-reducing behaviors. Modifiable behaviors (eg, exercise) remain poorly understood in the context of pain symptomology. We hypothesize that smart homes may assist with detecting behaviors that are influenced by pain (eg, sleep, socialization). However, data without contextual interpretation have little meaning in real-world situations and should be avoided in health care delivery [41,42]. Our smart home development methods illuminate one strategy for integrating clinical knowledge to support the development of a prototype pain-assessing smart home (PASH).

This multiple methods, secondary analysis used data from an ongoing longitudinal smart home study (2017-2021). The longitudinal study’s ML models have already demonstrated the ability to recognize 30 activities of daily living (eg, sleeping, eating, entering or exiting home) with approximately 98% accuracy based on 3-fold cross validation [43]. The longitudinal study aims to train an ML model to recognize changes in health states in real-time in older adults with comorbidities. Participants are monitored for 1-2 years using smart home sensors (passive infrared motion, magnetic door use, light, temperature, and humidity) that are deployed in their current residence. No cameras or microphones are used. Secured date and time-stamp sensor data are collected, and the ML model labels daily activities in real-time [44]. Additionally, expert nurses with advanced practice skills conduct weekly health assessments of participants via telehealth or home visit. Individualized semistructured interviews are conducted to obtain information about potential changes in health status and behavior, and any health-related concerns are documented. Participants are asked to recall health changes (ie, health events) occurring in the 7 days prior to the nursing visit. Participants are asked: “How has your health been in the last week?” and “Did you have any particular days that weren’t normal?” To elicit recall, questions are asked about each body system. For example, with a person having atrial fibrillation, the nurse might say: “I see your heart rate is X; have you had any issues with your heart since we last talked?” For sleep, they ask: “Were there any nights you didn’t sleep well? Or went to bed late, or got up early? What was different about that day?” This line of questioning is designed to elicit information about the “how, what, when, where, and why” of health events. Vital signs, information about medication changes, new symptoms by body system (eg, neuro, cardiac), sleep quality, psychosocial status, functional status, and changes to daily routines (eg, all-day outings) are captured. A participatory approach is encouraged, and most participants keep a daily journal to help with recall, though this is not required. Health events are documented and coded by medical type (eg, neuro, cardiac, respiratory). Nurses match health event and sensor data and interpret event start and stop times as well as pre-event and post-event activities and behaviors (ie, providing real-world context, clinical ground truth). To train PASH, we used these existing pain-related clinical and sensor-based data. More information about the role of nurses in the longitudinal study is available in the literature [42,45,46].

Although pain was not the focus of the longitudinal study (the focus was on chronic conditions), multiple episodes of pain were captured by nurses. These were available in the archived health assessment records and used for this secondary analysis. The purpose of this secondary analysis was to determine if ambient sensor-based data could be used to train an ML model that recognizes pain-related behaviors. The longitudinal study and secondary analysis were approved by the Washington State University Institutional Review Board.

## Methods

### Data Inclusion Criteria

To train an ML model to recognize pain-related behaviors, we conducted a secondary data analysis of historic longitudinal sensor-based data and semistructured, holistic nursing assessment interviews containing descriptions of pain. Historic records afforded a convenient and purposive sample [47] of 17 independent, community-dwelling smart home participants aged ≥55 years with ≥2 chronic conditions, living alone, without pets. Of these, 11 participants met the inclusion criterion of having at least one recorded pain event during the longitudinal study. For each participant, approximately 60,000 sensor events per month were available, totaling 720,000 per year. All data from passive infrared and door use sensors throughout 1-2 years’ monitoring were included for training the model. Based on prior work developing techniques to train ML models using small sample sizes [35,36], we determined this amount of data exceeds ML training requirements for models using longitudinal data and that this amount of data would likely allow the model to capture aspects of pain. The health records of *each* participant contained data from approximately 50-100 nursing assessments (1-2 years of weekly visits). We extracted pain-related information from the nursing record, defining a “pain event” as any report of pain associated with a report of related behavior changes. We also included 1 week of sensor data surrounding the pain event for conducting qualitative analysis of the sensor data as well as several weeks of baseline activity data — these were weeks where the nurse noted no health changes had occurred. “Normal” weeks were compared to weeks containing health events to help illuminate the event in the sensor data. We included data showing short-term visitors, which is exhibited in the data as multiple sensors turning ON nearly simultaneously (within <0.01 seconds of each other and <3 meters apart).

### Data Exclusion Criteria

We excluded personal health data unrelated to pain. When conducting qualitative analysis of the sensor-based data, we excluded sensor data outside of the week surrounding the pain event except the weeks chosen to represent baseline normal routines. We also excluded data showing extended stay visitors (ie, stays across multiple days and nights). More information on the nursing team’s analytic methods, including data exclusion processes, is available in the literature [45,46]. For training the ML model, we excluded data from light, temperature, and humidity sensors.

### Qualitative Analysis

#### Adapted Qualitative Descriptive Methods

We applied the Fritz method [45] when analyzing pain-related, sensor-based data to support our expert-guided approach. The Fritz method includes the parallel processing of qualitative health event data and associated sensor data for contextualizing health changes in sensor data, enabling the development of clinically accurate ground truth [45]. It is an analytic approach that uses qualitative data and traditions to make sense of sensor data. Nurses use subjective semistructured interviews, objective nursing assessments, medical records, and clinical knowledge of the human response to illness to understand participants’ health events and daily routines that are represented in the sensor data. For each identified health event, 1 week of sensor data around the time of the event are reviewed. Abnormal behavior patterns (not aligning with known daily routines) are identified and verified by comparing them with selected baseline routine datasets from the 6 months surrounding the health event. A clearly exhibited health event includes changes to normal routines (eg, wake time, time out of home, time in bathroom). For more information, see [45,46].

#### Analyzing Sensor Data

**Figure 1:**
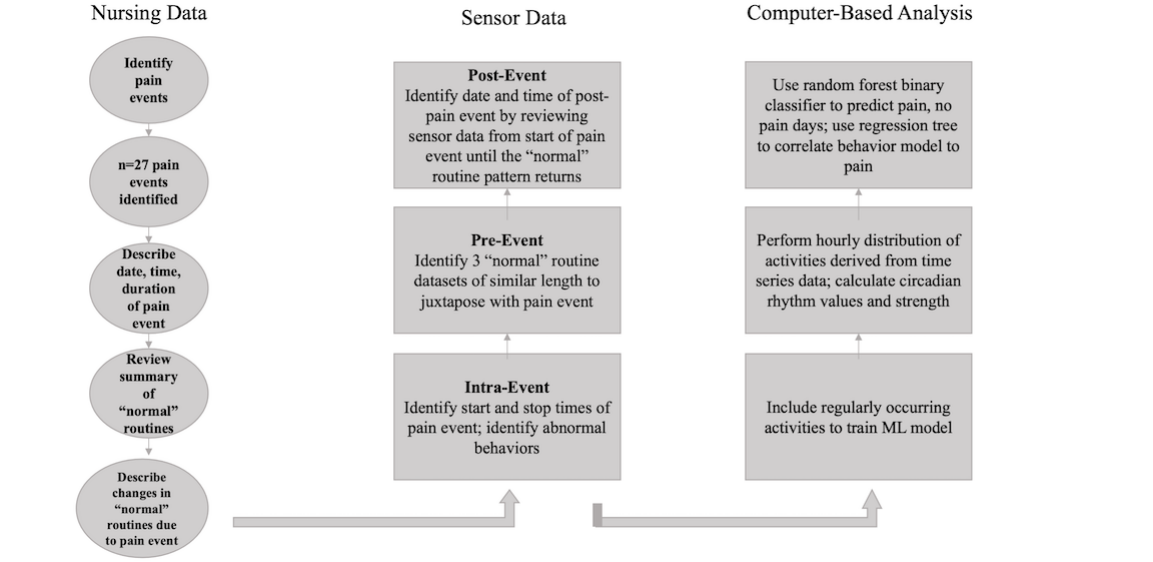
Multiple methods analytic processing of qualitative and quantitative data for a clinician-guided approach to machine learning (ML).

Two nurse analysts trained in qualitative descriptive methods [48] and the Fritz method [45] separately analyzed all 27 pain events. Each analyst used the nursing record, which included associated dates and times, diagnosis, and a summary of daily routines, to determine each pain event’s timing and activities. The first round of analysis was conducted by Nurse Analyst A as part of the ongoing longitudinal study. The second round of analysis was conducted by Nurse Analyst B for the current substudy. After the second round of analysis was complete, Nurse Analysts A and B met to discuss potential themes. Figure 1 illustrates the qualitative analytic process that preceded computer processing of the data.

To analyze each pain event, the nursing record was reviewed first; then, the document summarizing the participant’s daily routines was reviewed, and the associated sensor data were downloaded from a secure database. The day(s) of the event and a minimum of 1 week of sensor data surrounding the event (5 days before and 2 days after) were downloaded. If a pain event lasted 7 days, 2 weeks’ data were analyzed: the week-long event plus 5 days before and 2 days after. Additionally, a minimum of 3 weeks representing “normal/baseline” sensor data were downloaded. We defined a normal week as any week where (1) the nursing record reported the participant had a normal week (ie, they said nothing had changed, or they said they felt good), (2) overnight visitors were not present, and, (3) it was not a holiday. Nurse analysts then looked at activity timing and duration, activity sequences, and the amount of data produced by each sensor on the day(s) of the event. We determined the timing and duration of activities by observing the sensor label (eg, bed, recliner) and the time of day a sensor transition occurred (eg, between the bedroom and living room). We determined activity sequences by observing the order in which various sensors’ ON signals appeared. We determined the amount of data by calculating the total number of consecutive ON and OFF signals of a single sensor or cluster of sensors (eg, bed, general bedroom area). For example, insomnia or restlessness in the night was observed in the sensor data as an increase in the total number of consecutive bed sensor ON signals or the intermixing of other sensors’ ON signals (eg, kitchen sensors) instead of sensor quietness (ie, absence of sensor ON signals, representing sleep). Once the pain event was clearly identified in the sensor data (eg, significant change in sleep behavior), baseline data of routine behaviors from the surrounding weeks and months were compared to the behavior anomalies associated with the pain event. A minimum of 3 datasets per event representing normal routines were captured for comparison; however, nurse analysts continually expanded their review of the sensor data surrounding the pain event until they were satisfied that reviewing more data would not produce additional understandings.

#### Developing Themes

**Figure 2:**
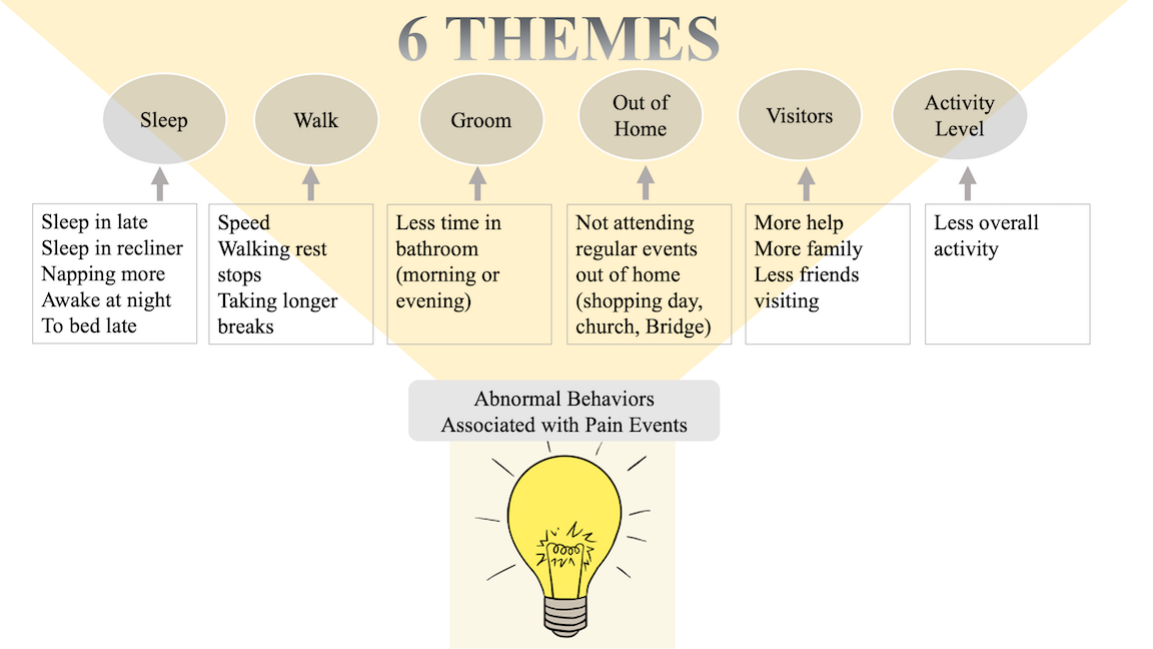
Abnormal pain-related behaviors observed in the sensor data, with 6 overarching themes.

Figure 2 illustrates the influence of abnormal behaviors (associated with experiencing pain) on emerging qualitative behavioral themes. Patterns in the sensor data representing pain-related behaviors became apparent when we compared sensor-based data across all 27 different pain events and across all participants (N=11). Emerging patterns were designated as behavioral themes. Some emerging themes incorporated related ideas; so, they were clustered together and assigned a larger, overarching idea that became the major theme. For example, the overarching idea of “Sleep” subsumed minor themes that regarded characteristics of sleep like the timing of sleep (eg, bedtime, wake time), length and quality of sleep (with and without interruption), and sleep location. Major themes were chosen based on 2 criteria: The theme was present across multiple events, and both nurses thought the theme was clinically relevant. No themes were dropped during the process of moving from minor to major themes. A final comprehensive re-review of transcripts did not reveal new themes. Both nurse analysts agreed on the chosen themes, and each nurse analyst’s ideas were equally valued and addressed. Major themes represent activities that persons experiencing pain will likely alter. Such knowledge, emerging from sensor-based observations and clinical interpretation, supported the training of PASH.

### Quantitative Analysis

The same 11 smart homes were included for both qualitative and quantitative analyses. Only 2 sensor types (passive infrared motion, door use) were processed for the quantitative portion of the study. Pain events varied in length from 1 to 15 days (mean 7.85 days); for each pain event, an equivalent set of relatively pain-free data was included for each participant’s pain event dataset. All collected smart home sensor events had already been automatically labeled with corresponding normal daily activities using our activity recognition algorithm that had previously been trained on 30 homes (not analyzed as part of this study). In each of these prior homes, a non-clinical annotator provided ground truth labels for 2 months of sensor data. From activity-labeled sensor data for the 11 homes included in this study, 550 behavior markers were extracted. A set of activities that occur with sufficient regularity in the homes (at least once a day on average) was selected to inform the ML models. The activity categories were Bathe, Bed-Toilet Transition, Cook, Eat, Enter Home, Leave Home, Personal Hygiene, Relax, Sleep, Take Medicine, Wash Dishes, Work, and Other Activity. The behavior markers corresponded to statistical measures of mean, median, standard deviation, maximum, minimum, zero crossings, mean crossings, interquartile range, skewness, kurtosis, and signal energy. Each measure was applied to time series data indicating hourly distributions for overall activity level (measured as the number of motion sensor events), hourly distributions over home locations, and hourly distributions over activity classes. Additionally, behavior markers were computed that indicate daily schedule regularity and 24-hour circadian rhythm values. The regularity value calculates the normalized difference (in location and in activity) between the same hours across all of the days in the sample. The circadian rhythm strength was calculated using a periodogram, which is an estimate of the spectral density of a signal (in this case, activity level). The periodogram identifies the strength of the frequencies that explain variations in time series data. We quantified circadian rhythm as the normalized strength of the frequency that corresponds to 24 hours.

We designed several ML approaches for detecting pain events from sensor-derived behavior markers. First, we employed a random forest binary classifier with 150 trees to predict whether a day was part of a pain event (positive class) or pain-free (negative class). Second, we trained a regression tree on the data to determine correlation between a model of behavior markers and pain events. For comparison with our clinician-trained ML approaches, we employed an isolation forest (iForest) anomaly detection algorithm with 100 estimators to detect pain days. Unlike the random forest and regression trees, iForest did not use any clinician guidance in detecting anomalies that may indicate days for which the participant was experiencing pain. Finally, we trained a decision tree algorithm based on the positive and negative instances to determine which behavior markers provided the greatest distinction between pain days and those that were pain-free. We did not train the algorithm to differentiate between pain subclasses due to the small number of events in each group (acute, flare); however, our clinical team’s early work determining these subgroups using sensor-based data positions us to explore subgroup comparisons in the future with larger sample sizes.

## Results

Table 1 shows the sample characteristics of the participants and number and duration of acute and flair pain events. A total of 11 older adult participants, aged 68-92 years, were included in this secondary data analysis. All participants were community-dwelling and living independently while being monitored with smart home sensors, except 1 participant who moved to assisted living during the study.

**Table 1:** Demographic characteristics of the study sample (N=11).

Characteristics		Results
Age (years), mean (range)		85.72 (69-92)
**Biologic sex, n (%)**		
	Female	9 (82)
	Male	2 (18)
**Marital status, n (%)**		
	Married	1 (9)
	Divorced	1 (9)
	Widowed	9 (82)
**Education, n (%)**		
	High school (some or graduated)	2 (18)
	College (some or graduated with Bachelor’s)	7 (64)
	Graduate School (Master’s or Doctorate)	1 (9)
**Independence, n (%)**		
	Living independently	11 (100)^a^
	Living alone	11 (100)
	Uses assistive personnel (excluding housekeeping)^b^	2 (18)
	Uses a housekeeper	10 (91)
	Uses assistive equipment^c^	4 (36)
**Pain event (n=27), n (%)**		
	Acute (duration=0.25-14 days; mean duration 6.8 days)	8 (30)
	Flare (duration=2-8 days; mean duration 6.6 days)	19 (70)

^a^One participant entered assisted living after 17 months in the study.

^b^Assistive tasks: donning compression stockings (independent participant), medication administration, showering (assisted-living participant).

^c^Equipment: 4-wheeled walker, electric scooter, prosthetics.

### Qualitative

We found 13 pain-related behaviors: no exit home, decreased time out of home, visitors, sleep location, time of sleep, length of sleep, night time sleep interruption, change in total sleep hours (increase or decrease), sleep quality (body movement during sleep — tossing and turning), grooming, walking speed, change in walking pattern (short bursts, long rests), overall activity in 24 hours*.* From the sensor activity patterns, 6 themes representing all 13 behaviors emerged (Figure 2): Sleep, Walking, Grooming, Time Spent Out of Home, Visitors, Overall Activity Level. Themes represented pain-related behaviors. Table 2 shows major and minor theme characteristics, the types of pain reported by participants (noted by nurses), and the type of sensor, location, and sensor combinations informing qualitative interpretations. Themes generally aligned with the 13 activity categories that the ML algorithm recognized, and each of the 6 themes was represented in the subset of 30 activities (from the prior homes) included for training PASH. For example, the qualitative theme of “Time Spent Out of Home” was represented in PASH’s ML model as “Enter Home” and “Leave Home.”

**Table 2:** Themes representing pain-related behaviors.

Themes (activity attributes)	Participant-reported pain	Nursing report of participants’ behaviors	Sensors informing themes	Meaningful sensor combinations	Sensor type
Sleep (rhythm, length, location, quality)	Neck, leg, knee, hip pain	“slept in,” “moved to recliner,” “increased nap time,” “awake in night”	General bedroom, bed, recliner	<Bed-Toilet>, <Recliner-Toilet>, <Bed-Recliner>, <Bed-Bedroom>, <Bed-Kitchen>	Passive infrared (PIR)
Walking (speed, rests, breaks)	Fall, leg, knee, hip pain; chest pain	“shortness of breath,” “resting more frequently when walking”	Bedroom, bed, recliner, toilet, hallway	<Recliner-Toilet>, <Bed-Toilet>, <Hallway-Hallway>	PIR
Grooming (done, not done)	Fall, abdominal pain	“has not showered for 2 days,”^a^ “help with grooming”	Bathroom, bathroom sink	<General Bathroom Area-Bathroom Sink> (Quantity and duration)	PIR
Time spent out of home	Fall, neck, leg, knee, abdominal pain	“didn’t go to Bridge night,” “didn’t go shopping”	Main entry	<Main Entry-Hallway>, <Absence of sensor events>	Magnetic contact (door use)
Visitors (Social, health workers)	Fall, neck, leg, abdominal pain	“now has home health,” “caregiver at bedtime,” “daughter visit for 3 days^a^ to help”	General living room, recliner, kitchen sink, hallway, bathroom sink	Any 2 sensors with virtually concurrent ON signals (<0.01 seconds apart) located greater than 10 feet apart	PIR
Overall activity level (in 24 hours)	Fall; leg, hip, abdominal pain; chest pain	“didn’t attend exercise class,” “didn’t feel like doing much, just laid on sofa,” “mostly in bed for 2 days”^a^	All sensors	Total number of sensor events in 24 hours, room activity length and variety; account for time out of home	PIR, magnetic contact (door use)

^a^Nursing record contained actual dates of participant-reported pain events.

Sleep was the leading pain-related behavioral theme, accounting for 6 of the 13 described behaviors. Of the 11 participants, 8 (having 22 of 27 pain events [81%]) reported sleep changes that were observed in the sensor data. Leading observable sleep behaviors included location (more time in a recliner), timing (bedtime, wake time, napping), hours of sleep at night, and sleep quality. Six of 8 acute pain events (short-term pain not associated with underlying pain) affected sleep, resulting in 2 people spending more time in their recliner chair, 1 person experiencing decreased overall sleep (day and night) and an inability to sleep in bed, and 3 people with restless sleep (decreased sleep quality). Twelve of 19 flare pain events (exacerbation of underlying pain) affected sleep, observed as more time in bed during the day, earlier bedtimes, more time spent in recliners, and intermittent changes in sleep location across several months. Two walking characteristics were observed: Walking speed slowed, and the number or length of rest breaks increased. We observed this in the monthly Timed-Up-and-Go tests and by calculating the difference in the time it took to move between specific sensors (bed and toilet or recliner and toilet).

Grooming activities were observed by reviewing bathroom sink, bedroom, and bedroom closet sensor groupings. A lack of grooming recorded in the nursing record as “stayed in bed 3 days on Dilaudid” was seen in the sensor data as a decrease in overall time spent in the bathroom in the mornings and evenings. A lack of grooming appeared to be associated with pain intensity. For example, the nursing record reported that 1 participant said, “I just haven’t felt like leaving the house or even showering. I mostly lay in bed all day.” Other pain-related behavior modifications regarded participants’ overall activity level (in 24 hours) and time spent out of the home. All acute pain events resulted in decreased overall activity: 2 reduced their out-of-home activities, and 2 had more visitors. Pain-event interpretations, event start and stop times, ground truth annotations, and themes were communicated to the computer science team to support the training of PASH.

### Quantitative

The random forest classifier that was used to distinguish pain from pain-free days yielded a 3-fold cross-validation classification accuracy of 0.70, sensitivity of 0.72, specificity of 0.69, area under the receiver operating characteristic curve value of 0.756, and area under the precision-recall curve of 0.777. We use the term pain-free to distinguish between pain events and routine days; participants may not have actually been pain-free. To check the impact of clinicians’ ground truth annotations and the expert-guided approach, we used standard anomaly detection techniques to determine classification accuracy without expert guidance. We used iForest to determine periods of time that were generally considered anomalous without clinician guidance. Using the detected anomalies (no clinician guidance) as indicators of pain events yielded a predictive accuracy of 0.16, a difference from the random forest (with clinician guidance) that was statistically significant (*P*<.001). Using the regression tree, a moderate correlation (*r*=0.415) was found between the behavior models and an indication of pain on the corresponding days.

The decision tree classifier created a greedy ordering of behavior features that best distinguish pain from pain-free days using information gain as the ordering criterion. These results highlighted 3 features that provided a high level of differentiation between the 2 classes: normalized overall activity level (lower for pain days), time spent in bed-toilet transition activities (higher for pain days), and time spent in a favorite chair (higher for pain days). Behaviors such as overall activity level, walking speed (or time spent in bed-toilet transition), and time spent in a favorite chair (including sleeping there) were supported by both qualitative and quantitative analytic findings and may prove important to understanding pain experiences.

## Discussion

### Principal Findings

Our findings show preliminarily that ambient sensor-based data can be used to train an ML model to recognize pain-associated behaviors. These findings align with previous studies that indicate ML algorithms are capable of detecting behaviors that indicate a clinically relevant change in health status [21,23,32,33,49,50]. Unlike these previous studies, which primarily focused on associating behaviors with cognitive and functional health, we focused on behaviors exhibited by persons experiencing pain. While there is significant overlap in pain-related behaviors and other known health-related behaviors that machines can recognize, we uniquely discovered that one behavior not typically included in pain interference scales (yet recognized as physical activity sequences by ML algorithms) point to the existence of increased pain: decreased grooming. Importantly, almost all the qualitative themes (emerging from sensor-based data) align with behaviors that are already well-established with validated pain interference scales [26]. The exception is “Grooming.” Grooming is not specifically identified as a factor in some of the most commonly used pain interference measurement scales (eg, the Brief Pain Inventory); yet, it could become an important target to assess pain populations when using sensor monitoring. This is an example of how smart home monitoring can generate new evidence-based information to support pain management.

Given that the standard anomaly detection techniques used to determine classification accuracy without expert guidance yielded a 16% classification accuracy compared with the expert-guided approach (predictive accuracy 70%), our findings support the ideas that (1) clinicians, such as nurses with frontline pain management experience, add value to the efficacy of the ML model and (2) PASH offers possibilities as a clinical tool for identifying pain-related behaviors. Though the model demonstrated a pragmatically low predictive accuracy (70%) for clinical applications and we did not ask it to discriminate between pain subclasses (acute, flare), it performed quite well given the small participant sample size (N=11) and small number of captured pain events (N=27). PASH’s accuracy could improve given a larger participant sample size and greater number of pain-related training events interpreted by clinicians.

The question of whether all pain leads to the same pain-related behaviors could be raised. Our smart home approach to pain management cannot yet determine the type of pain. Further, this approach to pain assessment cannot determine the source, severity, or location of a person’s pain (eg, abdominal versus head). However, given that ML models are capable of recognizing clinically relevant behavior changes [21,29], it is reasonable to consider that ML models could alert when anomalous pain-related behaviors occur regardless of which behavior the model chooses to prioritize or the pain characteristics. Based on our preliminary findings, it is also plausible that, with larger samples, ML models could be trained to alert on unrealized pain-related behaviors. Such alerts, based on naturalistic real-time data of persons experiencing and attempting to manage pain, would be of significant value to clinicians seeking to perform early interventions using minimal pharmacologics. ML affords this opportunity while also individualizing pain context and offering the potential to discover new perspectives on pain. Randomized controlled trials supporting current interventions do not account for individual differences in pain experiences. Rather, they focus on average pain responses, leaving persons who are outliers without optimal care. Learning how individuals with pain uniquely express their pain moment-by-moment could lead to novel understandings of pain and afford the opportunity to provide effective, precise interventions. To achieve such precision, PASH would benefit from adding ecological momentary assessment, an in-the-moment data capture technique for naturalistic settings [51] now regarded as the most accurate method for capturing real-world pain [52,53].

### Clinical Implications

PASH offers clinicians a more objective, data-driven way of knowing about pain. PASH could be of benefit to nonverbal or cognitively impaired individuals [54]. Developing consistent, reliable, objective pain measurements; detecting patterns in behaviors and activities that exacerbate or relieve pain; and accurately capturing responses to medications and other pain treatments are potential scientific discoveries that could be realized using an AI system like PASH. In addition, PASH could add new sensor-based evidence of biopsychosocial pain components and facilitate the combining of traditional and new data to augment and support clinical assessments (with reduced bias) and clinical decision making. We recognize that it is impossible to know when an acute pain condition may transition into chronic pain; yet, part of our enthusiasm about this work is the future possibility of detecting minor and important changes in this regard using sensor data. This would be an important contribution to pain science.

Privacy and data security have been identified as primary concerns for older adults considering the use of a smart home. As ubiquity of data collection expands to the home environment and is integrated into the delivery of health care, considerations for data security are needed so risks for data breaches are mitigated and identities of vulnerable persons, such as persons in pain, cannot be stolen or easily reconstructed.

### Bias

Clinicians and software developers can potentially introduce bias into ML models [5,14,55]. Assumptions and generalized perspectives regarding subpopulations and disease progression tendencies incorporated in a clinician’s belief structure (overtly or inadvertently) over time could impact the reporting of ground truth. Likewise, software developers’ assumptions (recognized or unrecognized) have potential to impact design approaches and perceived end-user wants and needs. To avoid algorithm bias, clinicians and computer scientists as well as study participants need to be a diverse group of humans [56]. To accomplish this, intentionality toward diversity in all aspects of the design loop is required.

### Limitations

This project was limited by the larger study’s design and the purposive sample size (number of participants, number and type of pain events; both convenience samples). Using historic datasets not specifically collected for discovering pain-related behaviors as well as the small numbers of participants and pain events limit generalizability and make the model susceptible to overfit. Reliance on participants’ weekly recall of health changes, including pain, limits accuracy of sensor data interpretations. Not all qualitative and quantitative components of this multiple methods, secondary analysis aligned. We do not make an attempt to differentiate between depression and pain-related behaviors in this study and acknowledge the synergy between these conditions. Significant time and effort are required for the expert-guided approach to ML, which potentially limits scalability. Larger and more diverse samples, a longitudinal design, and use of an ecological momentary assessment for data collection are needed. Using ML to identify and discriminate between pain phenotypes would be of benefit to providers and patients. PASH should be tested in a prospective study to identify true and false positive ratios. Additionally, PASH needs to be trained to accommodate a multiperson household.

### Conclusion

Innovative monitoring and treatment options are needed to support persons experiencing chronic pain, their caregivers, and the health care professionals working alongside them to improve their quality of life and health outcomes. Our findings suggest that smart homes using AI monitoring tools are well-positioned to become pragmatically useful at detecting clinically relevant pain-related behaviors or relevant changes in those behaviors. Using smart homes to provide automated pain assessment and intervention could alleviate some of the pressure on patients and clinicians working in the pain management health care arena. Concrete, objective data demonstrating how people with pain are affected and how they self-manage painful conditions at home could be used to deepen understandings and innovate solutions. Leveraging such technologies for health care delivery should be done intentionally, and clinicians should participate in technology development studies to interpret data, provide meaningful context, and illuminate meaningful use possibilities in all phases of development.

## Abbreviations

AI: artificial intelligence
iForest: isolation forest
ML: machine learning
PASH: pain-assessing smart home

## References

[1] Dahlhamer J, Lucas J, Zelaya C, Nahin R, Mackey S, DeBar L, Kerns R, Von Korff M, Porter L, Helmick C (2018). Prevalence of Chronic Pain and High-Impact Chronic Pain Among Adults - United States, 2016. MMWR Morb Mortal Wkly Rep.

[2] Dowell D, Haegerich TM, Chou R (2016). CDC Guideline for Prescribing Opioids for Chronic Pain — United States, 2016. MMWR Recomm. Rep.

[3] Darnall B, Juurlink D, Kerns R, Mackey S, Van Dorsten B, Humphreys K, Gonzalez-Sotomayor JA, Furlan AJ, Gordon AJ, Gordon DB, Hoffman DE, Katz J, Kertesz SG, Satel S, Lawhern RA, Nicholson KM, Polomano RC, Williamson OD, McAnally H, Kao MC, Schug S, Twillman R, Lewis TA, Stieg RL, Lorig K, Mallick-Searle T, West RW, Gray S, Ariens SR, Sharpe Potter J, Cowan P, Kollas CD, Laird D, Ingle B, Julian Grove J, Wilson M, Lockman K, Hodson F, Palackdharry CS, Fillingim RB, Fudin J, Barnhouse J, Manhapra A, Henson SR, Singer B, Ljosenvoor M, Griffith M, Doctor JN, Hardin K, London C, Mankowski J, Anderson A, Ellsworth L, Davis Budzinski L, Brandt B, Hartley G, Nickels Heck D, Zobrosky MJ, Cheek C, Wilson M, Laux CE, Datz G, Dunaway J, Schonfeld E, Cady M, LeDantec-Boswell T, Craigie M, Sturgeon J, Flood P, Giummarra M, Whelan J, Thorn BE, Martin RL, Schatman ME, Gregory MD, Kirz J, Robinson P, Marx JG, Stewart JR, Keck PS, Hadland SE, Murphy JL, Lumley MA, Brown KS, Leong MS, Fillman M, Broatch JW, Perez A, Watford K, Kruska K, Sophia You D, Ogbeide S, Kukucka A, Lawson S, Ray JB, Wade Martin T, Lakehomer JB, Burke A, Cohen RI, Grinspoon P, Rubenstein MS, Sutherland S, Walters K, Lovejoy T (2019). International Stakeholder Community of Pain Experts and Leaders Call for an Urgent Action on Forced Opioid Tapering. Pain Med.

[4] van Hartskamp M, Consoli S, Verhaegh W, Petkovic M, van de Stolpe A (2019). Artificial Intelligence in Clinical Health Care Applications: Viewpoint. Interact J Med Res.

[5] Topol E (2019). Deep Medicine: How Artificial Intelligence Can Make Healthcare Human Again.

[6] Odaibo D, Zhang Z, Skidmore F (2019). Detection of visual signals for pneumonia in chest radiographs using weak supervision. SoutheastCon IEEE.

[7] Kalpathy-Cramer J, Freymann J, Kirby J, Kinahan PE, Prior FW (2014). Quantitative Imaging Network: Data Sharing and Competitive AlgorithmValidation Leveraging The Cancer Imaging Archive. Transl Oncol.

[8] Regalia G, Onorati F, Lai M, Caborni C, Picard RW (2019). Multimodal wrist-worn devices for seizure detection and advancing research: Focus on the Empatica wristbands. Epilepsy Res.

[9] Rajpurkar P, Hannun AY, Haghpanahi M (2017). Cardiologist-level arrhythmia detection with convolutional neural networks.

[10] Clifford G, Liu C, Moody B, Lehman LH, Silva I, Li Q, Johnson AE, Mark AG (2017). AF classification from a short single lead ECG recording: The PhysioNet/computing in cardiology challenge 2017.

[11] Topalovic M, Das N, Burgel P, Daenen M, Derom E, Haenebalcke C, Janssen R, Kerstjens HA, Liistro G, Louis R, Ninane V, Pison C, Schlesser M, Vercauter P, Vogelmeier CF, Wouters E, Wynants J, Janssens W, Pulmonary Function Study Investigators (2019). Artificial intelligence outperforms pulmonologists in the interpretation of pulmonary function tests. Eur Respir J.

[12] Niel O, Boussard C, Bastard P (2018). Artificial Intelligence Can Predict GFR Decline During the Course of ADPKD. Am J Kidney Dis.

[13] Lemley KV (2019). Machine Learning Comes to Nephrology. JASN.

[14] Yang YJ, Bang CS (2019). Application of artificial intelligence in gastroenterology. World J Gastroenterol.

[15] Kumar N, Verma R, Sharma S, Bhargava S, Vahadane A, Sethi A (2017). A Dataset and a Technique for Generalized Nuclear Segmentation for Computational Pathology. IEEE Trans. Med. Imaging.

[16] Campanella G, Hanna MG, Geneslaw L, Miraflor A, Werneck Krauss Silva V, Busam KJ, Brogi E, Reuter VE, Klimstra DS, Fuchs TJ (2019). Clinical-grade computational pathology using weakly supervised deep learning on whole slide images. Nat Med.

[17] Roberts A, Gaizauskas R, Hepple M, Davis N, Demetriou G, Guo Y, Kola J, Roberts I, Setzer A, Tapuria A, Wheeldin B (2007). The CLEF corpus: semantic annotation of clinical text. AMIA Annu Symp Proc.

[18] Rehm G, Kuhn B, Lieng M, Cortes-Puch I, Nguyen J, Guo EC, Delplanque JP, Anderson NR, Adams JY (2019). An analytic platform for the rapid and reproducible annotation of ventilator waveform data. bioRxiv.

[19] Albright D, Lanfranchi A, Fredriksen A, Styler WF, Warner C, Hwang JD, Choi JD, Dligach D, Nielsen RD, Martin J, Ward W, Palmer M, Savova GK (2013). Towards comprehensive syntactic and semantic annotations of the clinical narrative. J Am Med Inform Assoc.

[20] Dawadi PN, Cook DJ, Schmitter-Edgecombe M (2013). Automated Cognitive Health Assessment Using Smart Home Monitoring of Complex Tasks. IEEE Trans. Syst. Man Cybern, Syst.

[21] Sprint G, Cook DJ, Fritz R, Schmitter-Edgecombe M (2016). Using Smart Homes to Detect and Analyze Health Events. Computer.

[22] Sprint G, Cook D, Fritz R, Schmitter-Edgecombe M (2016). Detecting health and behavior change by analyzing smart home sensor data.

[23] Rantz M, Skubic M, Abbott C, Galambos C, Popescu M, Keller J, Stone E, Back J, Miller SJ, Petroski GF (2015). Automated In-Home Fall Risk Assessment and Detection Sensor System for Elders. Gerontologist.

[24] Skubic M, Guevara RD, Rantz M (2015). Automated Health Alerts Using In-Home Sensor Data for Embedded Health Assessment. IEEE J. Transl. Eng. Health Med.

[25] Keogh A, Tully MA, Matthews J, Hurley DA (2015). A review of behaviour change theories and techniques used in group based self-management programmes for chronic low back pain and arthritis. Man Ther.

[26] Wilson M (2014). Integrating the concept of pain interference into pain management. Pain Manag Nurs.

[27] LeBaron V, Hayes J, Gordon K, Alam R, Homdee N, Martinez Y, Ogunjirin E, Thomas T, Jones R, Blackhall L, Lach J (2019). Leveraging Smart Health Technology to Empower Patients and Family Caregivers in Managing Cancer Pain: Protocol for a Feasibility Study. JMIR Res Protoc.

[28] Williams JA, Cook DJ (2017). Forecasting behavior in smart homes based on sleep and wake patterns. THC.

[29] Cook DJ, Schmitter-Edgecombe M, Dawadi P (2015). Analyzing Activity Behavior and Movement in a Naturalistic Environment Using Smart Home Techniques. IEEE J. Biomed. Health Inform.

[30] Sprint GL, Cook DJ, Fritz R (2020). Behavioral Differences Between Subject Groups Identified Using Smart Homes and Change Point Detection. IEEE J. Biomed. Health Inform.

[31] Debes C, Merentitis A, Sukhanov S, Niessen M, Frangiadakis N, Bauer A (2016). Monitoring Activities of Daily Living in Smart Homes: Understanding human behavior. IEEE Signal Process. Mag.

[32] Dawadi P, Cook D, Schmitter-Edgecombe M (2016). Automated Cognitive Health Assessment From Smart Home-Based Behavior Data. IEEE J Biomed Health Inform.

[33] Rantz MJ, Skubic M, Popescu M, Galambos C, Koopman RJ, Alexander GL, Phillips LJ, Musterman K, Back J, Miller SJ (2015). A New Paradigm of Technology-Enabled ‘Vital Signs’ for Early Detection of Health Change for Older Adults. Gerontology.

[34] Galambos C, Skubic M, Wang S, Rantz M (2013). Management of Dementia and Depression Utilizing In- Home Passive Sensor Data. Gerontechnology.

[35] Hu N, Englebienne G, Lou Z, Krose B (2017). Learning to Recognize Human Activities Using Soft Labels. IEEE Trans. Pattern Anal. Mach. Intell.

[36] Dahmen J, Cook D (2019). SynSys: A Synthetic Data Generation System for Healthcare Applications. Sensors (Basel).

[37] Mukasa D, Sung J (2020). A prediction model of low back pain risk: a population based cohort study in Korea. Korean J Pain.

[38] Lötsch J, Sipilä R, Tasmuth T, Kringel D, Estlander A, Meretoja T, Kalso E, Ultsch A (2018). Machine-learning-derived classifier predicts absence of persistent pain after breast cancer surgery with high accuracy. Breast Cancer Res Treat.

[39] Atee M, Hoti K, Hughes J (2018). A Technical Note on the PainChek™ System: A Web Portal and Mobile Medical Device for Assessing Pain in People With Dementia. Front Aging Neurosci.

[40] Meretoja TJ, Andersen KG, Bruce J, Haasio L, Sipilä R, Scott NW, Ripatti S, Kehlet H, Kalso E (2017). Clinical Prediction Model and Tool for Assessing Risk of Persistent Pain After Breast Cancer Surgery. JCO.

[41] Forsythe DE, Hess DJ (2001). Studying Those Who Study Us: An Anthropologist in the World of Artificial Intelligence.

[42] Dermody G, Fritz R (2019). A conceptual framework for clinicians working with artificial intelligence and health-assistive Smart Homes. Nurs Inq.

[43] Aminikhanghahi S, Cook DJ (2019). Enhancing activity recognition using CPD-based activity segmentation. Pervasive and Mobile Computing.

[44] Cook DJ, Crandall AS, Thomas BL, Krishnan NC (2013). CASAS: A Smart Home in a Box. Computer.

[45] Fritz RL, Dermody G (2019). A nurse-driven method for developing artificial intelligence in "smart" homes for aging-in-place. Nurs Outlook.

[46] Fritz RL, Cook D (2017). Identifying varying health states in smart home sensor data: An expert-guided approach.

[47] Palinkas LA, Horwitz SM, Green CA, Wisdom JP, Duan N, Hoagwood K (2015). Purposeful Sampling for Qualitative Data Collection and Analysis in Mixed Method Implementation Research. Adm Policy Ment Health.

[48] Neergaard MA, Olesen F, Andersen RS, Sondergaard J (2009). Qualitative description - the poor cousin of health research?. BMC Med Res Methodol.

[49] Cook DJ, Schmitter-Edgecombe M, Jonsson L, Morant AV (2019). Technology-Enabled Assessment of Functional Health. IEEE Rev Biomed Eng.

[50] Robben S, Englebienne G, Krose B (2017). Delta Features From Ambient Sensor Data are Good Predictors of Change in Functional Health. IEEE J. Biomed. Health Inform.

[51] Shiffman S, Stone AA, Hufford MR (2008). Ecological momentary assessment. Annu Rev Clin Psychol.

[52] Heapy A, Dziura J, Buta E, Goulet J, Kulas JF, Kerns RD (2014). Using multiple daily pain ratings to improve reliability and assay sensitivity: how many is enough?. J Pain.

[53] Carlozzi NE, Schilling S, Freedman J, Kalpakjian CZ, Kratz AL (2018). The reliability of end of day and ecological momentary assessments of pain and pain interference in individuals with spinal cord injury. Qual Life Res.

[54] Schiavenato M, Craig K (2010). Pain assessment as a social transaction: beyond the "gold standard". Clin J Pain.

[55] Yapo A, Weiss J (2018). Ethical implications of bias in machine learning. http://hdl.handle.net/10125/50557.

[56] Obermeyer Z, Powers B, Vogeli C, Mullainathan S (2019). Dissecting racial bias in an algorithm used to manage the health of populations. Science.

